# Editorial: Immunology of human fungal pathogens: host-pathogen interactions, immune evasion, and therapeutic strategies

**DOI:** 10.3389/fimmu.2026.2003290

**Published:** 2026-10-14

**Authors:** Iván Martínez-Duncker, Andrea Regina de Souza Baptista, Héctor M. Mora Montes

**Affiliations:** 1 Laboratorio de Glicobiología Humana y Diagnóstico Molecular, Centro de Investigación en Dinámica Celular, Instituto de Investigación en Ciencias Básicas y Aplicadas, Universidad Autónoma del Estado de Morelos, Cuernavaca, Mexico; 2 Center for Microorganism Research, Biomedical Institute, Fluminense Federal University, Rio de Janeiro, Brazil; 3 División de Ciencias Naturales y Exactas, Departamento de Biología, Universidad de Guanajuato, Guanajuato, Mexico

**Keywords:** fungal, immunology, infections, mycology, therapy

The interaction between pathogenic fungi and the immune system is remarkably dynamic. The fungal cell wall, once conceived primarily as a static protective barrier, is now recognized as a highly adaptable structure that changes in response to morphology, environmental conditions, antifungal exposure, and the transition from free-living growth to a parasitic lifestyle within the host (1). These adaptations are not merely structural: they determine which fungal molecules become visible to immune surveillance and consequently influence recognition, inflammation, persistence, and disease progression. Indeed, fungal pathogenesis emerges from a continuous dialogue between fungal adaptation and host immunity rather than from virulence or immunodeficiency alone.

An illustrative example is provided by *Fonsecaea pedrosoi*, in which the muriform morphology characteristic of chromoblastomycosis represents both a structural adaptation and an immunological challenge. Chen et al. showed that chitin accumulation in the cell wall of muriform cells, a cell morphology resistant to macrophage-mediated killing and linked to the chronicity of chromoblastomycosis, impairs NOD-like receptor family pyrin domain containing 3 (NLRP3) inflammasome activation, providing a mechanism by which fungal morphology may contribute to immune evasion and disease chronicity. This finding extends the broader concept that cell-wall composition participates directly in pathogenesis and antifungal resistance, and illustrates how remodeling of the fungal surface can alter the immunological information presented to the host (2).

The cell wall is simultaneously one of the richest sources of fungal pathogen-associated molecular patterns (PAMPs). Their recognition by pattern-recognition receptors (PRRs) initiates many of the innate mechanisms required for antifungal defense. Even though several PRRs have been described during *Sporothrix brasiliensis* immune sensing (3), the particular *in vivo* contribution of Dectin-1 and Dectin-2 is not well understood. In experimental sporotrichosis, Yoshikawa et al. demonstrated that Dectin-1 and Dectin-2 contribute to limiting dissemination in a murine model of *S. brasiliensis* infection and influence CD8+ T-cell and regulatory T-cell responses. Although interleukin-17 (IL-17) contributed to host survival and tissue integrity, receptor-mediated restriction of fungal burden was not primarily explained by IL-17 signaling. Trentin et al. further emphasize the species-specific structural diversity of the cell walls of major priority fungal pathogens, including *Aspergillus fumigatus*, *Candidozyma auris*, *Cryptococcus neoformans*, and *Candida albicans*, species listed as critical priority fungal pathogens by the World Health Organization. Such structural diversity has consequences extending from immune recognition and immune evasion to vaccine development and the identification of new antifungal targets.

The cell wall is not the only source of fungal PAMPs. Fungal nucleic acids are also recognized by PRRs and contribute importantly to antifungal immune activation. Liang et al. reviewed the recent findings on the roles of cytosolic nucleic-acid sensors and Toll-like receptors 3, 7/8, and 9 in the sensing of fungal nucleic acids. Humoral immunity adds another dimension to this recognition system. Pruksaphon et al. revisit the comparatively underexplored contribution of immunoglobulin M (IgM) to antifungal immunity. The multimeric architecture of IgM provides high-avidity recognition of fungal carbohydrate structures even in the absence of somatic hypermutation, while complement activation promotes opsonophagocytic clearance. Particularly intriguing observations in *C. neoformans* indicate that natural IgM may also modify capsule and cell-wall properties and inhibit titan-like cell formation. Thus, antibody-mediated fungal defense should not be considered simply an adjunct to cellular immunity, but as another mechanism capable of influencing fungal phenotype itself.

Anatomical context shapes fungal disease. Li et al.in a single-center study of 145 patients from East China, found *Fusarium* spp. as the leading cause of fungal keratitis. In an immunophenotyping subset comprising 12 patients and matched controls, aqueous humor displayed neutrophil predominance, an increased proportional representation of B cells, and reduced representation and function of T cells and natural killer cells. Exposure, inoculation route, and the local immune environment must therefore be considered together when interpreting disease.

The capacity of the immune system to recognize fungi does not, however, guarantee their elimination. Successful pathogens conceal, alter, or exploit molecular signals that would otherwise initiate protective immunity. Liu et al. provide a broad framework for understanding this process in invasive fungal infections. Surface-antigen masking, biofilm formation, metabolic adaptation, toxin production, and polysaccharide capsules allow major pathogens such as *C. albicans*, *A. fumigatus*, and *C. neoformans* to escape immune surveillance. In immunocompromised individuals, these mechanisms converge with abnormalities of NLRP3 signaling, impaired Th1 and Th17 responses, T-cell exhaustion, and increased expression of inhibitory checkpoints such as programmed cell death protein 1 (PD-1). The resulting phenotype is therefore not simply “immunosuppression,” but an unstable immunological state in which inadequate fungal clearance may coexist with damaging inflammation

Lei et al. performed single-cell ribonucleic acid (RNA) sequencing on lesional tissue from one patient with chromoblastomycosis and one healthy control, followed by validation in lesions from five patients and a murine model. They identified an expanded population of dysfunctional CD4+ T cells expressing PD-1, T-cell immunoglobulin and mucin domain 3 (TIM-3), and lymphocyte activation gene 3 (LAG-3). Their analyses suggested progression from naive T cells toward exhaustion within the chronically inflamed lesion, with monocytes and macrophages potentially contributing through persistent antigen presentation and inhibitory ligand-receptor interactions. These findings complement the structural observations in *F. pedrosoi* muriform cells (Chen et al.). Together they suggest that chromoblastomycosis chronicity cannot be attributed to a single fungal or host mechanism: fungal remodeling can attenuate innate recognition, while prolonged persistence can subsequently reshape adaptive immunity toward exhaustion.

At mucosal surfaces, the same host-fungus interaction can produce a very different outcome. In vulvovaginal candidiasis, disease is strongly associated with immunopathology rather than with a simple failure to recruit inflammatory cells. Qian et al. review how *C. albicans* adhesins and secreted or membrane-associated factors—including secreted aspartyl proteases, agglutinin-like sequence proteins, candidalysin, lipases, and phospholipases—interact with vaginal epithelial and innate immune responses. Activation of inflammatory pathways by secreted aspartyl proteases and candidalysin-induced epithelial signaling promote cytokine and chemokine production and vigorous neutrophil recruitment. In this setting, therefore, immune activation contributes not only to fungal control but also to the clinical manifestations of disease.

This interplay between fungal persistence and immunopathology is particularly relevant to recurrent vulvovaginal candidiasis (RVVC), where manipulating host responses may eventually complement direct antifungal treatment. Theodoropoulou reports preliminary observations in four atopic patients with RVVC and *Candida* sensitization who received *Candida*-specific sublingual immunotherapy. Three patients were reported to have complete resolution of RVVC at follow-up, whereas one relapsed and required a longer course. The very small, selected cohort and uncontrolled design preclude conclusions regarding efficacy, but the study is conceptually provocative: in carefully defined forms of fungal-associated inflammatory disease, therapeutic success may ultimately involve recalibrating an inappropriate immune response rather than simply intensifying antifungal exposure.

The balance between protective and pathological immunity is also influenced by the ecological context in which fungi reside. Liu et al. provide an example of the role of fungal communities in shaping immune activation through analysis of the gut mycobiota in children with allergic rhinitis and functional constipation. They analyzed the gut mycobiota of 19 pediatric cases of allergic rhinitis with functional constipation and compared this group with 17 healthy individuals. Compared with healthy individuals, the affected group showed depletion of several fungal genera (*Cenococcum*, *Dentiscutata*, *Ambispora*, and *Saccharomyces* genera) and an ecological shift in interkingdom interactions associated with immunoglobulin E sensitization.

Extending the potential systemic influence of the mycobiome, Zhang et al. investigated *Kazachstania pintolopesii* in naturally aged mice and found that exposure to *K. pintolopesii* aggravated splenic senescence-associated phenotypes, accompanied by immune-cell reprogramming, iron accumulation, chronic inflammation, and ferroptosis-related changes. Astragaloside IV ameliorated several of these abnormalities in the experimental model. Although these observations remain preclinical, they suggest that fungi may affect systemic immune homeostasis beyond overt infection and show the value of integrated molecular analyses.

Beyond the ecological influences of the mycobiome, intrinsic characteristics of the host can profoundly determine the outcome of fungal exposure. Host genetic variation can produce paradoxical phenotypes in which apparently enhanced signaling coexists with deficient antifungal control. Gu et al. showed that the STAT1-L351F gain-of-function variant was associated with hyper-responsive interferon signaling yet impaired control of *Talaromyces marneffei*, illustrating how excessive activation of one immune pathway can coexist with defective antifungal defense. In the experimental model, this phenotype was accompanied by reduced natural killer T-cell frequencies and broad immune remodeling involving dysregulated myeloid/neutrophil effector responses and altered IL-17-related programs. These findings underscore that effective antifungal immunity depends not on generalized immune activation, but on the appropriate balance and coordination of interconnected immune pathways.

Whereas genetic immune dysregulation can predispose otherwise apparently immunocompetent individuals to fungal disease, acquired immunosuppression similarly increases susceptibility and can produce unusual clinical presentations. Inceli et al. reported an angioinvasive *A. flavus* infection causing a subclavian mycotic aneurysm and massive hemoptysis in a child with acute lymphoblastic leukemia. Molecular identification contributed to the diagnosis, while recovery followed urgent surgical resection and vascular reconstruction combined with dual antifungal therapy, illustrating the importance of multidisciplinary management.

Wang et al. describe an invasive deep-tissue infection caused by traumatic inoculation of *Neoscytalidium dimidiatum* in a patient with poorly controlled diabetes. An organism more commonly associated with superficial skin and nail disease produced rapidly progressive tissue necrosis. Antifungal susceptibility testing yielded relatively low minimum inhibitory concentration for several agents, including voriconazole, which was recommended as systemic therapy. Despite surgical debridement and continuous vacuum sealing drainage, the infection progressed and ultimately required hallux amputation. Following amputation, continued vacuum sealing drainage and wound care were associated with favorable healing without recurrence. The case illustrates the challenges of managing invasive infections caused by uncommon molds for which standardized therapeutic regimens and established susceptibility breakpoints are unavailable.

Mucormycosis represents an even clearer intersection between fungal virulence and host susceptibility. Zhong et al. characterized the local immune landscape of pulmonary mucormycosis by integrating transcriptomic and proteomic analyses of infected and matched non-infected lung tissues obtained from patients undergoing surgical resection. Although transcriptomic data indicated activation of genes associated with PRRs, cytokines, chemokines, iron metabolism, and innate and adaptive immune pathways, proteomic profiling revealed surprisingly limited induction of corresponding immune proteins and broad suppression of host-defense pathways. This transcriptome–proteome discordance suggests that apparent immune activation at the transcriptional level may fail to translate into a corresponding functional protein response at the site of infection, providing a potential molecular dimension to the ineffective host defense characteristic of this aggressive mycosis.

The clinical consequences of this aggressive infection are also illustrated by Sun et al., who report pulmonary mucormycosis caused by *Rhizopus oryzae* in a six-year-old girl with diabetes. Metagenomic next-generation sequencing of bronchoalveolar lavage fluid enabled rapid etiological identification. Primary isavuconazole therapy, together with glycemic control, reduced and localized the pulmonary lesion sufficiently to permit lobectomy, followed by recovery. Although an individual case cannot establish comparative or first-line efficacy, it demonstrates the potential value of integrating rapid molecular diagnosis, antifungal therapy, correction of host risk factors, and surgical source control in invasive mycoses.

Importantly, immune competence should not itself exclude fungal disease. Wang et al. report histoplasmosis with mediastinal lymphadenopathy in a immunocompetent municipal water department employee following substantial occupational exposure. Although the presentation initially resembled bacterial pneumonia or sarcoidosis, urinary antigen, serology, and lymph-node histopathology supported the diagnosis despite negative bronchoalveolar lavage studies. Histoplasmosis should therefore remain in the differential diagnosis of compatible respiratory syndromes in immunocompetent patients with meaningful exposure even outside traditionally recognized endemic regions.

Recognizing when fungal recovery represents disease rather than colonization constitutes another clinical challenge. Machado et al. examined *Trichosporon asahii* recovered from urine of hospitalized patients, many of whom were critically ill or catheterized. Positive cultures were frequently accompanied by leukocyturia, hematuria, and proteinuria, supporting the presence of a local inflammatory environment. Nevertheless, the authors appropriately emphasize that these associations cannot definitively distinguish colonization from active infection. All-cause mortality was high—14 of 19 patients (73.7%)—but could not be attributed specifically to *T. asahii*. Under the study’s disk-diffusion criteria, all isolates were susceptible *in vitro* to fluconazole and voriconazole. The authors advocate early recognition and timely susceptibility-guided therapy when infection is clinically suspected, although the study did not directly evaluate treatment timing or efficacy.

Wu et al. evaluated 127 patients with detectable *Pneumocystis jirovecii* to distinguish infection from colonization. Patients with infection exhibited higher fungal burden and a characteristic immune profile comprising increased inflammatory mediators, including IL-6 and IL-10, together with lymphopenia and reductions in CD4+ T cells and regulatory T cells. A model combining pathogen and host variables achieved an area under the receiver operating characteristic curve of 0.852 in the internal validation; pathogen burden, IL-10, and absolute lymphocyte count emerged as the most informative predictors. These findings illustrate the value of applying computational approaches to integrate microbiological and immune dimensions.

In a retrospective single-center cohort of 1,835 critically ill patients Gao et al. found invasive pulmonary aspergillosis (IPA) in 7.1% of patients. Among 208 patients with mycological evidence of *Aspergillus*, bronchoalveolar lavage galactomannan showed the highest sensitivity, whereas combinations involving serum galactomannan with culture or molecular testing increased specificity. Antifungal therapy and tracheostomy were associated with improved survival, although the design precludes causation.

Beyond determining whether infection is present, understanding how fungal virulence factors interact with specific host tissues is essential for explaining organ tropism and injury. This is particularly evident in *C. neoformans*, which can affect the lungs and the central nervous system. In the latter, it can cause meningoencephalitis, an infection with a high mortality rate. Ramesh et al. used human neural stem cells, neurons, and cerebral organoids to study the effects of glucuronoxylomannan and found reductions in synaptophysin, with susceptibility related to cellular phosphatidylcholine composition. Munzen et al. further showed that cryptococcal phospholipase B1 is critical for biofilm formation, capsule properties, and cryptococcoma formation in the mouse brain. These studies reinforce the concept that fungal molecules simultaneously shape immune recognition, tissue tropism, cellular injury, and persistence.

Antifungal drugs remain indispensable, but drug resistance, toxicity, limited antifungal classes, and poor outcomes in severely immunocompromised patients increasingly expose the limitations of a purely pathogen-directed strategy. One pragmatic approach is optimization of available antifungal combinations. In a retrospective, non-randomized, single-center cohort, Zhang and Huang found that voriconazole–caspofungin combination therapy was associated with better clinical and inflammatory outcomes than voriconazole alone in patients with chronic obstructive pulmonary disease and IPA. Host status remains central to such therapeutic decisions: Sun et al. identified immunocompromise as a risk factor for early-onset IPA in patients with viral pneumonia, while Hu et al. reviewed the potential complementary role of immune and fungal biomarkers in supporting timely diagnosis and prognosis in pulmonary aspergillosi.

Beyond drug combinations, restoration of phagocyte function is particularly attractive because neutrophils and macrophages are central effectors against many invasive fungi. Mattos et al. tested granulocyte–macrophage colony-stimulating factor (GM-CSF) in immunosuppressed mice with systemic multidrug-resistant *C. auris* infection. High-dose GM-CSF monotherapy improved survival and reduced fungal burden in several organs while increasing macrophage and neutrophil populations and enhancing fungal uptake and killing through reactive oxygen species and neutrophil extracellular traps. These findings provide experimental support for augmenting host immunity as an adjunct to antifungal treatment, although clinical translation will require defining which patients have an immune defect amenable to such stimulation.

More broadly, Liu et al. propose an integrated therapeutic framework in which direct antifungal therapy is combined with host-directed immunomodulation. Candidate strategies include cytokines such as GM-CSF and interferon-γ, immune-checkpoint modulation, monoclonal antibodies, vaccines, and adoptive cellular approaches. Vaccine development remains an especially active area in medical mycology. Rabacal et al. evaluated the NXT-2a pan-fungal vaccine strategy, based on a Kex1-derived consensus peptide from *P. jirovecii*, *A. fumigatus*, *C. albicans*, and *C. neoformans*, and demonstrated protective efficacy against experimental *Pneumocystis* infection in drug-immunosuppressed macaques.

Together, these approaches represent a shift from asking only which drug is active against a fungus toward asking which combination of pathogen-directed and host-directed interventions is appropriate for a particular fungal species, immune phenotype, anatomical site, and stage of infection. Also, it requires a more holistic integration of the immune response to fungal infection. It is rarely simply “too weak.” Excessive inflammasome activity can promote tissue injury, mucosal neutrophilic inflammation can drive symptoms, checkpoint pathways may restrain damaging inflammation even while contributing to T-cell exhaustion, and restoration of immunity may precipitate inflammatory complications. Consequently, indiscriminate immune stimulation could be as harmful as insufficient immunity. The goal of antifungal immunotherapy should therefore be immune correction rather than immune amplification.

Technological advances are increasing our knowledge on the fundamental pathgenic mechanisms of fungal infection and are driving precision therapeutic approaches increasingly conceivable. Single-cell RNA sequencing, as illustrated in chromoblastomycosis (Lei et al.), can identify dysfunctional immune populations that remain hidden in bulk tissue analyses. Integrated transcriptomics and proteomics, as demonstrated in pulmonary mucormycosis (Zhong et al.), can expose the difference between transcriptional activation and functional immune output. Dynamic immune phenotyping, pathogen genomics, spatial transcriptomics, and functional phagocyte assays could eventually identify whether an individual patient requires enhancement of innate killing, restoration of exhausted adaptive immunity, control of pathological inflammation, or conventional antifungal therapy alone (Liu et al.).

Precision antifungal care will require identifying the pathogen and the patient’s immune state, then selecting direct antifungal treatment and, where evidence supports it, a targeted host-directed intervention to modulate the immune response.
